# Characteristics of maize cultivars in Africa: How modern are they and how many do smallholder farmers grow?

**DOI:** 10.1186/s40066-017-0108-6

**Published:** 2017-03-17

**Authors:** Tsedeke Abate, Monica Fisher, Tahirou Abdoulaye, Girma T. Kassie, Rodney Lunduka, Paswel Marenya, Woinishet Asnake

**Affiliations:** 1 CIMMYT-Kenya, PO Box 1041-00621, Nairobi, Kenya; 2 CIMMYT-Ethiopia, c/o ILRI Sholla Campus, CMC Road, PO Box 5689, Addis Ababa, Ethiopia; 3 Present Address: College of Agricultural Sciences, Oregon State University, Corvallis, OR, USA; 4 IITA-Nigeria Ibadan, PMB 5320, Oyo Road, Ibadan, Nigeria; 5 ICARDA Sub-Saharan Africa Program, Addis Ababa, Ethiopia; 6 CIMMYT-Zimbabwe, PO Box MP163, 12.5 km Peg Mazowe Road, Harare, Zimbabwe

**Keywords:** Variety turnover, Age of varieties, Maize adoption, Smallholder agriculture

## Abstract

**Background:**

Maize is the most important cereal and most widely cultivated staple that plays a key role in the food security of sub-Saharan Africa (SSA). Although some countries have achieved significant gains in maize productivity, the SSA average yields are far below what could be obtained with improved cultivars under good crop management. Low cultivar turnover is one among many contributing factors to low maize yields in SSA. At present, there is a critical knowledge gap on the identity, number, and age of maize cultivars currently grown by smallholder farmers on the continent.

**Results:**

This study revealed that nearly 500 maize cultivars were grown in 13 African countries surveyed in the 2013/2014 main crop season. Sixty-nine percent of the cultivars each occupied <1% of the total maize area; only two cultivars occupied >40% and four occupied >30% area. Approximately 32% of all the cultivars were hybrids, 23% were improved open-pollinated varieties (OPVs), and 46% were locals. Eastern Africa (EA) and southern Africa (SA) accounted for about 43 and 38%, respectively, of all the cultivars reported, whereas West Africa’s (WA) share was 19%. The average area planted to modern cultivars in the surveyed areas was estimated at 57%—with EA, SA, and WA estimates of 82, 55, and 36%, respectively; however, increased adoption was not necessarily always related to improved productivity, as the latter depends on many additional factors. Each household planted an average of 1.781 cultivars (range 1–8). The overall weighted average age of the cultivars was 15 years, with hybrids and OPVs being 13 and 18 years, respectively.

**Conclusions:**

Maize variety turnover in SSA is slower than what is practiced in the USA and other world regions such as Latin America and Asia. The substantial variations among regions and countries in all parameters measured suggest a tailored approach to mitigation interventions. Findings of this current study pave the way for replacing the old cultivars with more recent releases that are tolerant or resistant to multiple stresses and are more resilient.

## Background

With some 36 million ha harvested annually, maize occupies the largest land area of all staples in SSA; annual maize grain production is estimated at nearly 72 million metric tons (MT).^1^ According to the 2011–13 FAOSTAT data, maize has the highest per capita calorie consumption in SSA of 348 kcal/person/day, followed by rice, 341; wheat, 245; cassava, 193; millet, 119; sorghum, 112; yams, 109; plantains, 68; groundnut, 48; and beans, 45. Maize is not only a strategic crop for the 48 countries that cultivate it here; more than 208 million people in SSA depend on maize for food security and economic well-being. Current yields of maize in SSA (estimated at 1.8 MT/ha) are far below the average potential that can be achieved with improved cultivars and crop management, even though some countries have been making significant productivity gains in recent years [1]. The low yield of maize in SSA is attributed to slow variety turnover, among many other constraints, resulting in low adoption of modern cultivars^2^ (MCs) [2–5].

Since its introduction into Africa in the sixteenth century [6], maize has been grown under a wide range of agroecologies and socioeconomic conditions. The status of maize as a strategic food security crop took prominence, especially following the devastating droughts of the early 1980s in eastern and southern Africa. Modern breeding and selection of maize in Africa have been going on since as early as the first decade of 1900s in Zimbabwe [7] and in the 1940s in Malawi [8], but significant improved cultivar development efforts started in the 1950s and 1960s.

**Fig. 1:**
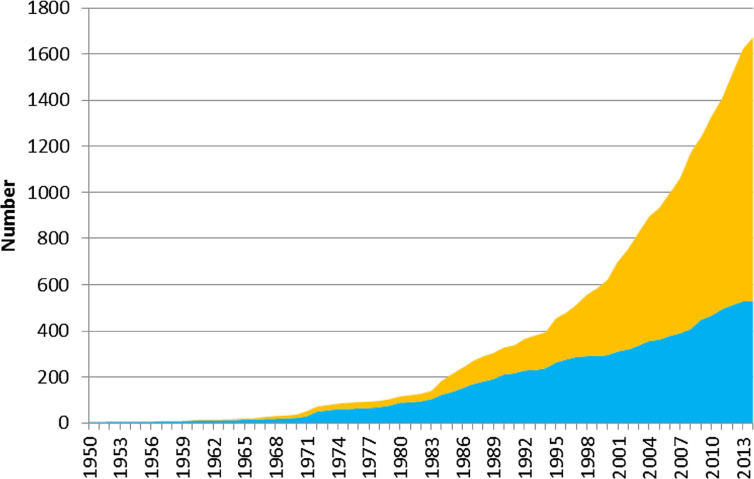
Running totals of maize cultivars released in SSA between 1950 and 2014 (*source*: own surveys) (consisting of 24 countries: Angola, Benin, Botswana, Burkina Faso, Cameroon, Chad, Cote d’Ivoire, DR Congo, Ethiopia, Ghana, Kenya, Lesotho, Malawi, Mali, Mozambique, Nigeria, Senegal, South Africa, Swaziland, Tanzania, Togo, Uganda, Zambia, and Zimbabwe.). *Orange shade* hybrids; *cyan shade* OPVs

Many countries in SSA have since developed and released significant numbers of cultivars. From the literature, nearly 1700 maize cultivars have been released between 1950 and 2014 (Fig. 1) across 24 countries; these consisted of roughly 68% hybrids and 32% OPVs. Accelerated variety release frequency has been observed over the last 15 years. For example, 64% of all the varieties were released in the 15 years between 2000 and 2014, compared to 36% in the preceding five decades. In other words, the annual rate of releases increased from 12 cultivars per year over the period from 1950 to 1999, to 73 cultivars per year during the period from 2000 to 2014. However, few countries in SSA have systems to track the status of maize cultivars currently grown.

Brennan and Byerlee [9] used weighted average age to determine the rate of wheat variety replacements. The literature on maize cultivars in SSA largely deals with adoption levels [10–15]. Other sources [16–20] mention number of cultivars released through a certain period and their adoption levels, but there is a scarcity of information on the actual number of cultivars grown and their turnover. This study presents detailed accounts of the status of maize cultivars that smallholder farmers grow at present across SSA. The specific contributions of this study are twofold. First, we summarize recent trends in the adoption of maize varieties across a wide range of countries into a unified summary. It is often the case that surveys such as these are limited to one country or regions within countries. Our coverage of 13 countries in eastern, western, and southern Africa represents a major contribution in bringing cross-continental data into one study, thereby providing the research and development community with a breath of data in a single study. Second, where the data were available, we summarized the vintage of the maize cultivars grown. The purpose was to assess the degree to which the seed systems in these regions are dynamic with regard to varietal renewal and replacement, a critical requirement to ensuring that the genetic gains from maize crop improvement programs are sustained and safeguarded. The focus on adoption and varietal replacement represents two critical elements of ensuring that the R&D efforts in maize breeding have sustained impacts on on-farm productivity as well.

## Methods of data collection

**Fig. 2:**
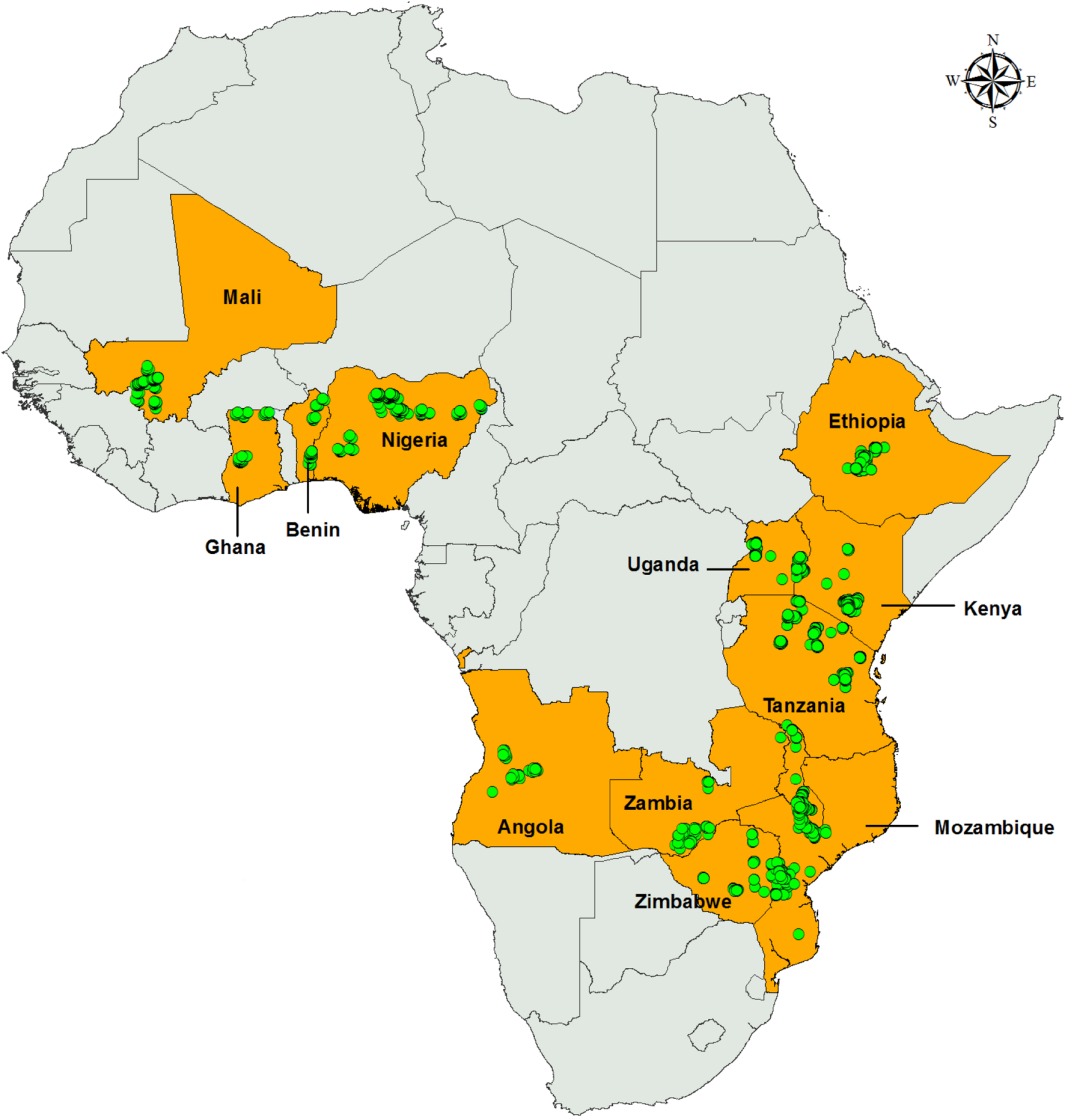
Study sites in the 13 surveyed African countries in the 2013/2014 main crop season. *Green dots* represent GPS coordinates of areas where sampling was done

The data for this report come from adoption monitoring household surveys carried out in 13 countries across SSA under the Drought Tolerant Maize for Africa (DTMA) project [5]. These adoption surveys were conducted in the main crop season of 2013 (except Mozambique, which was done in 2014). Sample sizes for the surveys ranged from 397 to 947 farm households per country, representing a total of 130 districts, 740 villages, and 7670 households (Table 1), depending on the area of maize cultivation in the country. The sampling sites are presented in Fig. 2.

**Table 1:** Number of districts, villages, and households sampled for adoption monitoring studies of drought-tolerant maize cultivars in the 2013/2014 maize season

Region/country	# districts	# villages	# households
EA totals	40	250	2500
Ethiopia	10	60	600
Kenya	10	60	600
Tanzania	10	90	900
Uganda	10	40	400
SA totals	50	260	2871
Angola	10	40	450
Malawi	10	40	595
Mozambique	10	60	626
Zambia	10	60	600
Zimbabwe	10	60	600
WA totals	40	230	2299
Benin	10	40	400
Ghana	10	60	555
Mali	10	40	397
Nigeria	10	90	947
SSA totals	130	740	7670

Sample selection was done in four stages. The first two stages were purposive. First, the main geographic areas and agroecologies where significant dissemination of DTMA seed had occurred were targeted in each country. Second, four to nine districts were selected within each target area, particularly districts where significant DT maize dissemination had occurred. The next two sampling stages were random. Ten villages were randomly selected in each selected district. Then, ten maize-farming households in each sampled village were randomly selected for interviews. Farm interviews, using a five-page questionnaire, were conducted between April and August of 2013 and in July 2014 (Mozambique). The short questionnaire, experienced enumerators, and close supervision by several authors of this paper ensured collection of high-quality data.

In each country, we took names of cultivars and the proportions of plots of each cultivar mentioned by each household. To determine the age of each cultivar, we referred to national and regional catalogues as well as personal contacts with relevant scientists, including ourselves, and compiled the release year. It will be seen in the sections below that there are large numbers of cultivars for which release years are not provided in almost all the 13 countries, but more commonly in West Africa.

The cultivars were then divided into their respective classes of hybrids, improved open-pollinated varieties (OPVs), and local (farmers’, traditional, or obsolete) cultivars. Our definitions of the different categories are as follows:

Hybrid: freshly purchased hybrid seed;
OPV: seed that has not been recycled for more than three seasons; and
Local (or farmers’ or traditional) cultivars: includes landraces, recycled hybrids, OPVs recycled more than three seasons, and or those for which no information is available on year of release.

## Results

### The cultivars

In this section, we provide an overview of the total number of cultivars grown across the 13 countries where the surveys were conducted, adoption rates of MCs, and their weighted average age. The list of total number of cultivars and other details for each country is available at www.stma.cimmyt.org. A total of 497 cultivars were reported (Table 2). Nearly half of those did not have information on YOR. The proportion of cultivars without YOR was roughly 47% in EA, 51% in SA, and 54% in WA. Overall, hybrids, OPVs, and local cultivars accounted for roughly 32, 23, and 46%, respectively (Table 2).

**Table 2:** Number of different classes of maize cultivars grown during the 2013/2014 main crop season in the 13 surveyed African countries

Region/country	Numbers				Without YOR		Covering <1% area	
	Hybrid	OPV	Local	Total	Number	Percent	Number	Percent
EA totals/avg.	69	58	87	214	101	47.2	154	72.0
Percentages	32.2	27.1	40.7	100.0				
Ethiopia	10	5	7	22	5	22.7	7	31.8
Kenya	33	33	18	84	33	39.3	68	81.0
Tanzania	17	12	54	83	55	66.3	66	79.5
Uganda	9	8	8	25	8	32.0	13	52.0
SA totals/avg.	79	15	93	187	95	50.8	122	65.2
Percentages	42.6	8.0	49.5	100.0				
Angola	4	2	25	31	24	77.4	20	64.5
Malawi	15	6	9	30	10	33.3	17	56.7
Mozambique	7	3	31	41	33	80.5	28	68.3
Zambia	30	2	27	59	27	45.8	42	71.2
Zimbabwe	23	2	1	26	1	3.8	15	57.7
WA totals/avg.	9	39	48	96	52	54.2	65	67.7
Percentages	9.4	40.6	50.0	100.0				
Benin	0	8	8	16	8	50.0	14	87.5
Ghana	5	13	19	37	23	62.2	27	73.0
Mali	0	8	2	10	2	20.0	0	0.0
Nigeria	4	10	19	33	19	57.6	24	72.7
SSA totals/avg.	157	112	228	497	248	49.9	341	68.6
Percentages	31.7	22.5	45.8	100.0				

The composition of cultivars for SA was roughly 43% hybrids, 8% OPVs, and 50% locals, whereas in EA it was 32% hybrids, 27% OPVs, and 41% locals. By contrast, hybrids accounted for just over 9%, OPVs for 41%, and local cultivars for 50% in WA. On average, the area covered under MCs in the surveyed areas was about 82% in EA, 55% in SA, and 36% in WA of total maize area planted to all maize cultivars (Table 2).

Twenty-eight of the cultivars were grown in two to five countries each during the 2013/2014 main crop season. Most notable among those were DK8053 (reported from Kenya, Tanzania, Malawi, Mozambique, and Zambia); Obatanpa (Ghana, Mali, Nigeria, Uganda, and Zambia); PAN53 (Malawi, Mozambique, Zambia, Zimbabwe, and Ghana); SC513 (Tanzania, Malawi, Mozambique, Zambia, and Zimbabwe); DK8031 (Kenya, Uganda, Malawi, and Mozambique); ZM521 (Ethiopia, Angola, Malawi, and Zimbabwe); PAN67 (Kenya, Tanzania, Mozambique, and Zambia); and SC627 (Tanzania, Malawi, Zambia, and Zimbabwe). Seven of the 28 cultivars were reported from three countries each, and 13 were reported from two countries each. Obatanpa was the only cultivar that was grown in all the three regions. It is known by its original name Obatanpa in Ghana and Zambia; in Mali, it is called Dembanyuman; it is Sammaz14 in Nigeria; and in Uganda, it goes by the name Longe5 (or Nalongo). Katumani was the oldest cultivar grown in three countries: Kenya (released in 1967), Tanzania (released in 1968), and Ethiopia (released in 1974). Hickory King, released in 1909, was the oldest improved maize cultivar on record in SSA; it occupied 1.7% of all maize area in the surveyed localities of Zimbabwe in 2013.

**Fig. 3:**
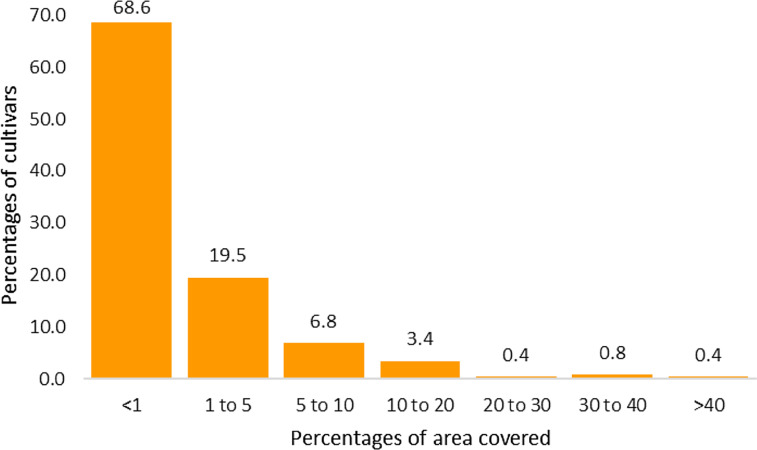
Relationship between percentages of maize cultivars and area covered in the 13 surveyed African countries in the 2013/2014 main crop season

The maize cultivars scene in Africa is dominated by many cultivars covering small areas, as illustrated in Fig. 3: For example, nearly 69% of the cultivars recorded in the 2013/2014 main crop season across SSA each occupied less than 1% of the total area. By contrast, only two of the cultivars each occupied more than 40% of the total area. Only four cultivars occupied more than 30% of the total maize area. Cultivars with very high area coverage in our survey included Obatanpa in Ghana (41%), SC513 in Zimbabwe (40%), Kanyani in Malawi (38%), Branco Redondo in Angola (37%), BH540 in Ethiopia (36%), and Longe5 in Uganda (35%). Sotubaka in Mali (24%), SC Duma43 in Kenya (20%), and Cagolo in Mozambique (18%) were also worth mentioning.

De Groote et al. [21] reported that no individual cultivar occupied more than 10% of the total maize area in Zambia in the 2011 crop season. By contrast, Smale and Olwande [19] reported that the hybrid H614D occupied 43% of all the modern maize plots planted by farmers in Kenya in 2010.

The top five ranked maize cultivars grown in selected countries across the three regions during the 2013/2014 main crop season and related details are presented in Table 3. The area occupied by the highest cultivar in each country ranged from 10% in Zambia to nearly 41% in Ghana. The area covered under the top five ranking cultivars was also highly variable among countries; slight differences were also observed among regions. Overall, the average area occupied by the top five ranked cultivars ranged between 11% for Benin and 79% for Zimbabwe (average = 49%). The regional averages for SA and EA were roughly 57% each, whereas the WA average was 32% (Table 3). This is a significant improvement over the adoption rates recorded by Morris [13] for 1997. His report showed MCs adoption of 34% for SSA, 36% for EA, 32% for SA, and 10% for WA.

**Table 3:** Top five ranked maize cultivars grown in the 13 surveyed African countries during the 2013/2014 main crop season

Region/country	Varieties (descending order of area coverage)	Area covered (%)			Year of release	
		Highest	Lowest	Total	Oldest	Latest
	EA avg.	25.6	5.8	56.6	20.3	7.3
Ethiopia	BH540^a^, Shone^a^, Agar^a^, Melkassa2^b^, Katumani^c^	35.5	4.3	65.6	1974	2008
Kenya	SC Duma43^a^, DHO2^a^, WS505^b^, Kikamb^a^, DK8031^a^	19.9	7.4	47.6	1995	2004
Tanzania	Staha^b^, Situka1^b^, DK8031^a^, PAN67^a^, TMV1^b^	12.0	5.2	38.7	1983	2002
Uganda	Longe5^a^, Longe10H^a^, Longe4^b^, Longe6H^a^, Longe 7H^a^	35.2	6.2	74.6	2000	2009
	SA avg.	28.7	5.8	57.4	27.0	14.6
Angola	Branco Redondo^c^, Amarelo^c^, Catete^c^, Nanhala^c^, Vermelho^c^	36.8	5.3	74.0	1957	1967
Malawi	Kanyani^a^, PAN53^a^, Makangala^a^, ZM309^b^, Bantum^a^	37.8	6.7	65.2	1999	2009
Mozambique	Cagolo^c^, Chimanhica^c^, PAN67^a^, Bantamo^c^, Chindau^c^	18.1	7.5	27.8	NA	NA
Zambia	PAN53^a^, MRI624^a^, SC513^a^, ZMS606^a^, MRI634^a^; Gankata^a^	10.4	5.2	40.9	1999	2006
Zimbabwe	SC513^a^, PAN413^a^, PHB3253^a^, SC403^a^, SC627^a^	40.3	4.4	79.0	1993	1999
	WA avg.	22.1	2.1	32.3	32.5	8.0
Benin	INA^c^, DMR^c^, Faaba^b^, SG2000^c^, TZPB SR^b^	13.5	1.5	11.3	1989	196
Ghana	Obatanpa^b^, Aburohoma^b^, Yegboni^c^, Appiah,^c^, Aburotia^b^	40.6	2.7	48.2	1983	1992
Mali	Sotubaka^b^, Dembanyuman^b^, Burkina^c^, Nieleni^b^, Zangreni^b^, N'Boni^a^	23.5	2.9	47.0	1985	1998
Nigeria	Oba Super 9^a^, EVDT 99^b^, 3DT Com^b^, Yar Masara^c^	10.8	1.3	22.7	NA	2009
	SSA avg.	25.7	4.7	49.4	26.6	10.3

a Hybrid

b OPV

c local

Table 4 provides the average and maximum number of maize cultivars grown by the sampled households in each study country. The numbers varied from region to region and from country to country. The overall average number of cultivars grown in the 2013/2014 crop season across the surveyed areas in the 13 countries was 1.781 per household (range 1–8 cultivars). The EA region had the highest average number of cultivars per household (1.969), followed by WA (1.724) and SA (1.677). Among the countries, Nigeria had the highest average number of 3.363 cultivars per household, followed by Kenya and Tanzania, with 2.527 and 2.135 cultivars per household, respectively. Mali, with 1.149 cultivars per household, had the lowest average number of cultivars grown (Table 4).

**Table 4:** Number of maize cultivars grown per household, by country in 2013/2014

Region/country	Average	95% confidence interval	Range
EA avg.	1.969	–	[1, 5]
Kenya	2.527	[2.475, 2.580]	[1, 5]
Tanzania	2.135	[2.086, 2.185]	[1, 4]
Uganda	1.904	[1.835, 1.974]	[1, 4]
Ethiopia	1.308	[1.267, 1.350]	[1, 3]
SA avg.	1.677	–	[1, 8]
Zambia	1.816	[1.721, 1.911]	[1, 8]
Mozambique	1.836	[2.559, 1.113]	[1, 5]
Zimbabwe	1.729	[1.673, 1.784]	[1, 5]
Angola	1.577	[2.015, 1.138]	[1, 5]
Malawi	1.429	[1.379, 1.479]	[1, 4]
WA avg.	1.724	–	[1, 4]
Nigeria	3.363	[3.330, 3.395]	[2, 4]
Ghana	1.220	[1.169, 1.271]	[1, 4]
Benin	1.162	[1.119, 1.205]	[1, 4]
Mali	1.149	[1.111, 1.186]	[1, 3]
SSA avg.	1.781	–	[1, 8]

In Zambia, the number of cultivars grown per household ranged from 1 to 8; in Kenya, Mozambique, Zimbabwe, and Angola, it was from 1 to 5; in Nigeria, it was from 2 to 4; in Tanzania, Uganda, Malawi, Ghana, and Benin, it was from 1 to 4, whereas it was from 1 to 3 cultivars per household in Ethiopia and Mali (Table 4). More details follow in the section dealing with each country, farther below.

### Age of cultivars

The weighted average age of maize cultivars grown during the 2013/2014 main crop season is presented in Table 5. The average age of hybrid maize cultivars for SSA was 13 years, whereas OPVs were more than 18 years old, with the overall weighted average of all cultivars being 15 years. Likewise, the overall average age of all cultivars was 14 years in EA, 15 years in SA, and 16 years in WA. These varied substantially from country to country (see later sections for more detailed discussions).

**Table 5:** Weighted average age of maize cultivars grown in the 13 surveyed African countries during the 2013/2014 main crop season

Region/country	Hybrids	OPVs	All
EA avg.	13.0	15.2	13.8
Ethiopia	10.6	17.7	13.5
Kenya	13.7	12.4	13.2
Tanzania	14.2	22.0	17.2
Uganda	10.7	16.4	13.4
SA avg.	12.4	26.5	15.4
Angola	NA	36.0	36.0
Malawi	10.7	10.1	10.3
Mozambique	10.5	16.0	11.9
Zambia	12.8	13.5	12.8
Zimbabwe	13.4	57.5	16.9
WA avg.	13.0	16.8	16.4
Benin	NA	10.4	10.4
Ghana	6.0	24.0	22.7
Mali	NA	17.9	17.9
Nigeria	14.8	11.8	12.6
SSA avg.	13.0	18.1	14.9

### Adoption rates

Table 6 depicts adoption levels of maize cultivars in the 13 countries included in the survey during the 2013/2014 main crop season. The overall SSA average adoption rate for hybrids and OPVs was roughly 37 and 21%, respectively. This meant that 57% of the total maize area in the surveyed areas of SSA was planted to modern maize cultivars in the 2013/2014 main crop season. Furthermore, 24% of the total maize area in SSA was under named local cultivars. Unidentified local cultivars occupied more than 18% of the total maize area across the continent; however, there were appreciable variations among regions and countries within them (see later sections for more detailed discussions).

**Table 6:** Area covered under different seed classes of maize in the 13 surveyed African countries during the 2013/2014 main crop season

Region/country	Modern cultivars			Local cultivars		
	Hybrids	OPVs	Total	Named	Unidentified	Total
EA avg.	52.2	29.4	81.6	10.1	8.3	18.4
Ethiopia	66.0	11.3	77.3	11.1	11.7	22.7
Kenya	65.0	17.1	82.1	17.5	0.5	17.9
Tanzania	40.2	31.7	71.8	9.4	18.7	28.2
Uganda	37.6	57.6	95.2	2.4	2.5	4.8
SA avg.	50.3	4.7	55.0	38.6	6.4	45.0
Angola	4.1	1.5	5.6	89.5	4.8	94.4
Malawi	65.7	12.8	78.5	14.6	6.9	21.5
Mozambique	24.9	5.1	30.0	70.0	0.0	70.0
Zambia	61.5	2.3	63.8	18.5	17.7	36.2
Zimbabwe	95.4	2.0	97.5	0.2	2.4	2.5
WA avg.	3.7	32.3	36.0	20.6	43.5	64.1
Benin	0.0	12.8	12.8	28.6	58.7	87.2
Ghana	3.1	50.3	53.4	25.5	21.1	46.6
Mali	0.0	51.2	51.2	13.1	35.7	48.8
Nigeria	11.6	14.7	26.3	15.4	58.4	73.7
SSA avg.	36.5	20.8	57.3	24.3	18.4	42.7

In summary, there were substantial variations among the three regions and countries within them in all the parameters measured in this study.

### Eastern Africa

EA accounted for the largest number of maize cultivars, 214 (or 43% of the total) in SSA. These consisted of 69 hybrids, 58 improved OPVs, and 87 local cultivars; 103 (48%) of the cultivars did not have information on YOR. The proportions of cultivars without YOR ranged from roughly 32% each for Ethiopia and Uganda to 65% in Tanzania (cf. Table 2). Seventy-two percent of the cultivars occupied less than 1% of the total area each. This ranged between nearly 32% in Ethiopia to 81% in Kenya. The weighted average age of hybrids and OPVs here was 13 and 15 years, respectively, with the overall average of MCs being about 14 years (cf. Table 5). Each household in EA grew an average of 1.969 cultivars (range 1–5), as presented in Table 4. The total adoption rate of MCs was roughly 82% (consisting of 52% hybrids and 29% OPVs), the highest among the three regions. Roughly 10 and 8% of the area was covered under local and unidentified cultivars, respectively (cf. Table 6).

### Ethiopia

The total number of maize cultivars grown in Ethiopia during the 2013 main crop season was 22 (cf. Table 2). These consisted of ten hybrids, five OPVs, and seven local cultivars. Five of those (or 23%) did not have information on YOR. In addition, nearly 32% of the total cultivars reported covered less than 1% of the total area each. The intermediate maturity group hybrid BH540 occupied the largest area (nearly 36%). This cultivar was released in 1995 and currently due for replacement, with recently released more robust and higher-yielding cultivars such as BH661 and BH546. Shone, Agar, Melkassa2, and Katumani were among the top five cultivars mentioned by farmers (cf. Table 3). Katumani (released in 1974) was the second oldest cultivar and occupied more than 4.33% of the total area in the 2013 main crop season. Shalla (released in 2011) and Melkassa6Q (drought-tolerant and QPM cultivar released in 2008) were also mentioned by the farmers interviewed, but they both covered less than 1% of the total area. The majority (86%) of households in Ethiopia grew one cultivar, whereas 12 and 2%, respectively, grew two and three cultivars. The average number of maize cultivars grown per household in the surveyed area of Ethiopia in the 2013 crop season was 1.308 (cf. Table 4). By comparison, Beshir and Wegary [22] reported up to seven maize cultivars planted per household in the Central Valley between 2001 and 2010.

The total maize area planted to MCs was about 77%—consisting of 66% hybrids and 11% OPVs; named and unidentified local cultivars occupied 11 and 12%, respectively (cf. Table 6).

These adoption figures are much higher than what had been reported for Ethiopia in the past. Byerlee and Jewell [10] and Maredia et al. [11] reported MCs adoption of 13–29% in 1990. Morris [13] reported approximately 4% of the area in Ethiopia to be under hybrid maize in 1997. Spielman et al. [14] reported that the area covered by improved seed was 2% in 1995 and 20% in 2003. Langyintuo et al. [23] reported coverage by MCs of about 18%. De Groote et al. [24] reported MCs adoption of 28% for national average and 18% for the Central Rift Valley in 2009. Jaleta et al. [25] reported MCs adoption of 31% during the 2011 main crop season. A recent study by Abate et al. [1] showed that the area covered by MCs jumped from 13% in 2004 to 40% in 2013.

The weighted average age of hybrids and OPVs in Ethiopia was roughly 11 and 18 years, respectively, with the overall average of 14 years (cf. Table 5). The OPV A-511, released in 1973, was the oldest cultivar that was being grown in 2013, but it occupied less than 1% of the maize area.

### Kenya

Kenya had the largest number of maize cultivars (84) grown among all the countries included in this study (cf. Table 2). These comprised 33 hybrids, 33 OPVs, and 18 local cultivars. Thirty-nine percent of the cultivars did not have information on YOR. The Seed Co hybrid Duma43 occupied the largest area (nearly 20%), followed by DH02, WS505, Kikamba, and DK8031. These five cultivars had combined total area coverage of nearly 48% (cf. Table 3). By contrast, 81% of the cultivars occupied less than 1% of the total area each. Each household planted an average of 2.527 cultivars (range 1–5), the highest in the EA region (cf. Table 4). Roughly 27% of the households planted one cultivar; 43% planted two cultivars; 22% planted three cultivars; 7% planted four cultivars; and 1% planted five cultivars in the 2013 main crop season in Kenya.

The weighted average age of hybrids was 14 years, whereas OPVs were 12 years old, with the combined average of 13 years. These figures are lower than the 18 years reported by Smale and Olwande [19] for 2010. The oldest cultivars reported in the current survey were Katumani Composite (2.74% area) and H511 (0.16%), both released in 1967.

The total area under MCs was 82%, consisting of 65% hybrids and 17% OPVs (cf. Table 6); less than 18 and 1% were under named and unidentified local cultivars, respectively. The high percentages of maize area covered by MCs reported here are consistent with several reports in the past. For example, Byerlee and Jewell [10] and Maredia et al. [11] reported 62% area under hybrids and 70% under MCs, whereas Morris [13] reported 65% area under hybrids and less than 8% under OPVs in 1997. However, the current area under OPVs is significantly greater than what was reported by Maredia et al. [11] for 1990, 1996, and 2006. De Groote et al. [26] reported a 69% MCs adoption rate in 2009. Smale and Olwande [19] reported 83% of households in Kenya planted hybrid maize in 2009–2010.

### Tanzania

Like Kenya, Tanzania also had a wide diversity of maize cultivars under production during the survey. A total of 83 cultivars were mentioned in the current surveys. These included 17 hybrids, 12 OPVs, and 54 local cultivars (cf. Table 2). A total of 55 cultivars (or 66%) did not have information on YOR. Dominant cultivars included Staha, Situka 1, DK8031, PAN67, and TMV 1 (cf. Table 3). These occupied a combined area of nearly 39%. The drought-tolerant OPVs Vumilia K1 (released in 2009) and ZM623 (released in 2007) were also mentioned by respondents, but their area coverage was less than 1% for both. Katumani (1.05% area) and H511 (0.16%) were the oldest cultivars reported; they were both released in 1968. Westengen et al. [27] reported that Staha and TMV1 accounted for two-thirds of all maize fields in the Morogoro area of Tanzania. Nearly 80% of all the cultivars reported covered less than 1% of the total maize area each (cf. Table 2).

Hybrids occupied 40% of the total area, whereas 32% were occupied by OPVs. The area covered under named and unidentified local cultivars was 9 and 19%, respectivley (cf. Table 6). This suggests that the total area occupied by MCs was nearly 72%. These figures are much more optimistic than previous reports. Byerlee and Jewell [10] and Maredia et al. [11] reported 6% of the area covered by hybrids. Morris [13] reported 6% area under hybrids and 4% under improved OPVs, with the remaining 90% planted to farm-saved seed. Lyimo et al. [18] reported maize area estimates under MCs of 26% in the 2009/2010 crop season. These figures are similar to what was reported by Langyintuo et al. [23].

The majority (51%) of households grew one cultivar, whereas 33% grew two cultivars, 11% grew three cultivars, and 5% grew four cultivars. The average number of cultivars grown per household was 2.135 (cf. Table 4).

The average age of maize cultivars currently in production in Tanzania was 14 years for hybrids and 22 years for OPVs. The oldest cultivars reported during this survey were the OPV Katumani and the hybrid H511, both released in 1968. They covered just over 1.05 and 0.13%, respectively.

### Uganda

The survey in Uganda revealed that 25 maize cultivars were planted in the 2013 main crop season. These comprised nine hybrids, and eight each of OPVs and local cultivars (cf. Table 2). Thirty-two percent of the total cultivars reported did not have information on YOR. In addition, 13 cultivars (or 52%) occupied less than 1% of the total maize area each in the 2013 main crop season. The OPV Longe5 (Nalongo^3^) occupied the largest proportion of maize area in Uganda (cf. Table 3). This was followed by Longe10H, Longe4, Longe6H, and Longe7H. These five cultivars occupied roughly 75% of the total maize area in the 2013 main crop season; all of these were released between 2000 and 2009. Recently released cultivars mentioned by farmers during this study included the drought-tolerant cultivars, Longe9H, and Longe11H. Longe10H (a drought-tolerant cultivar released in 2009) is already the most widely cultivated hybrid in Uganda.

Hybrids occupied approximately 38%, whereas OPVs occupied 58%; with named and unidentified cultivars covering the remaining 4% (cf. Table 6). This meant that the total area occupied by MCs was 95%. This is a significant improvement over the 10% area under hybrids in 1990 [10, 11]; 32% under MCs in 1996 [12]; 5% under hybrids and 50% under OPVs in 1997 [13]; and 14% hybrids and 21% under OPVs reported by Langyintuo et al. [23].

Sixty percent of farmers in Uganda grew one cultivar, whereas 29, 9, and 2%, respectively, grew two, three, and four cultivars in the 2013 main crop season. The average number of cultivars grown per household was 1.904 cultivars (cf. Table 4).

The weighted average age of hybrids in Uganda was 11 years, whereas that for OPVs was 16 years, with the overall weighted average of 13 years. The oldest cultivar reported in Uganda was Kawanda Composite, released in 1971; it accounted for 4.84% of the total maize area in 2013.

### Southern Africa

The SA region was home to a total of 187 maize cultivars (38% of the SSA total) that comprised 79 hybrids, 15 OPVs, and 93 local cultivars (cf. Table 2). Nearly 43% of the cultivars were hybrids, with OPVs and local cultivars comprising 8% and nearly 50%, respectively. Ninety (or nearly 51%) of the total cultivars had no information on YOR. A little over 65% of the cultivars covered less than 1% of the total area (cf. Table 2). The average number of cultivars grown per household was 1.677 (range 1–8), as presented in Table 4. The average maize area under hybrids here was a little over 50%, with nearly 5% OPVs. Thus, the total maize area under MCs was about 55%, with named and unidentified cultivars occupying about 39% and a little over 6%, respectively (cf. Table 6). The average area of the top five ranked cultivars was a little over 57% (cf. Table 3).

The maximum number of maize cultivars grown per household varied from five for Mozambique and Zambia, to four in Angola and Zimbabwe, and three in Malawi.

The weighted average age of hybrids, OPVs, and combination of both was roughly 12, 27, and 15 years, respectively. There were appreciable variations among countries within this region, in terms of number of cultivars, adoption rate, and age of cultivars.

### Angola

A total of 31 cultivars, consisting of four hybrids, two OPVs, and 25 locals were reported in the 2013 main crop season survey. Twenty-four (a little over 77%) of those were without YOR, and nearly 65% of all the cultivars covered less than 1% of the total maize area (cf. Table 2). Branco Redondo, Amarelo, Catete, Nanhala, and Vermelho were the top five ranked cultivars; together, these cultivars covered 74% of the total maize area in Angola. All of these were released between 1957 and 1967 (cf. Table 3).

Angola was the only country in SA that has not shown improvement in MCs adoption over 1997. Less than 6% of the total maize area was planted to MCs (4.1% hybrids and 1.5% OPVs) in the 2013 main crop season, whereas the area planted to named and unidentified local cultivars was a little less than 90 and 5%, respectively (cf. Table 6). With the exception of R1, farmers could not specifically name the hybrids; they said one of the hybrids was provided by Catholic Relief Services (CRS, an NGO), and another was from a Brazilian company named SEDIAC, that is based in Huambo province [28]. The OPVs ZM521 and ZM623 were the only modern cultivars mentioned by the farmers, but they occupied a little over 1% each. Nearly 37% of the total area surveyed was occupied by Branco Redondo (released in 1967 and now categorized as a local variety); this was followed by Amarelo (released in 1959) and Catete (released in 1957). Available literature (e.g., [12, 13, 23]) on adoption of MCs in Angola has shown little or no progress; it was even worse than what it was 20 years ago.

The average number of maize cultivars per household in 2013 was 1.577 (cf. Table 4). Half of the maize-growing households in Angola planted one cultivar, whereas 43% planted two; 6% planted three cultivars; and 1% planted four cultivars.

Part of the explanation for low or little adoption of modern maize cultivars in Angola was that there have been no companies marketing maize in this country until recent times. Currently, there are three seed companies—Fazenda Mato Grosso, Fazenda Agropecuária, and SEDIAC, all of which were established between 2004 and 2006.

The average age of known maize cultivars (all of which were OPVs and local cultivars) in Angola was 36 years.

### Malawi

A total of 30 maize cultivars were reported from Malawi in the 2013 main growing season. These included 15 hybrids, six OPVs, and nine local cultivars (cf. Table 2). Information on YOR could not be found for a third of the total cultivars reported; in addition, a little less than 57% of all the cultivars occupied less than 1% of the total maize area each in the 2013 main crop season. The hybrid Kanyani (SC403), released in 1999, was the dominant cultivar and occupied nearly 38% of the total maize area in the 2013 main crop season. This was followed by PAN53, Makangala, ZM309, and Bantum (cf. Table 3). Together, these five cultivars covered a little over 65% of the total maize area. With close to 7% of the total maize area in 2013, ZM309 was the most widely cultivated drought-tolerant OPV in Malawi; it was released in 2009. ZM523 and ZM623, both drought-tolerant cultivars, were among the top ten cultivars also mentioned by farmers; the former was released in 2009, whereas there was no YOR for the latter—released very likely between 2000 and 2006. Chokonaka (or MH18) and MH17 were the oldest cultivars (both released in 1991) reported during this study. The two accounted for 2.52 and 2.18%, respectively. The average number of maize cultivars planted per household in the 2013 main crop season in Malawi was 1.429 (range 1–4), as given in Table 4. Approximately 58% of the farmers in Malawi planted one cultivar, whereas 35, 6 and 1% grew two, three, and four cultivars, respectively.

The total maize area occupied by MCs was nearly 79% (consisting of nearly 66% hybrids and 13% OPVs); named and unidentified local cultivars accounted for a little less than 15 and 7%, respectively (cf. Table 6). Available literature (e.g., [10–12, 23]) indicates Malawi has made good progress with its maize adoption rates over the last two decades—the area under MCs was just over 11% in 1997. Malawi has among the shortest age of cultivars among the countries surveyed in this study. The weighted average age of hybrids, OPVs, and combined averages of the two was 11, 10, and 10 years, respectively (cf. Table 5).

### Mozambique

The number of maize cultivars reported in Mozambique in the 2014 main crop season was 41 (cf. Table 2). These comprised seven hybrids, three OPVs, and 31 local cultivars. No information on the YOR could be found for nearly 81% of the total cultivars; a little over 68% of the total cultivars occupied less than 1% each of the total maize area. The local cultivar Cagolo occupied about 18% of the total area; it was followed by Chimanhica, PAN67, Bantamo, and Chindau; together, these cultivars occupied nearly 28% of the total area (cf. Table 3). PAN53, released in 2011, was the only modern drought-tolerant hybrid that was being adopted in the 2014 main crop season. The drought-tolerant OPV ZM309 was also reported during this survey, but it occupied 0.16% of the total maize area. The average number of maize cultivars grown per household in Mozambique in the 2014 crop season was 1.836 (range 1–5) (cf. Table 4). Mozambique is one of the countries where smallholder households in the 2013 main crop season grew up to five cultivars each. This included 40% growing one cultivar; 41% growing two; 15% growing three; 3% growing four; and 1% growing five.

The total area covered by MCs was 30% (consisting of roughly 25% hybrids and 5% OPVs), with the remaining 70% being under local cultivars; there were no unidentified cultivars in Mozambique in the 2014 main crop season (cf. Table 6). According to available literature [10–13, 23], Mozambique has made very slow progress in MCs adoption over the last two decades—the 1997 adoption of MCs was 8%—compared to Malawi and Zambia.

The weighted average age of maize hybrids in Mozambique was roughly 11 years, whereas OPVs were 16 years old, with a combined average age of 12 years. Matuba, released in 1982, was the oldest known cultivar grown in Mozambique during the 2014 main season; it occupied approximately 4.6% of the total area.

### Zambia

Zambia had the third largest number of maize cultivars grown in the 2013 main crop season, after Kenya and Tanzania. A total of 59 maize cultivars were reported here; these consisted of 30 hybrids, two OPVs, and 27 local cultivars (cf. Table 2). Information on the YOR was not found for 27 (nearly 46%) of the total cultivars; 42 (or 71%) of the total cultivars occupied less 1% of the total maize area each grown in the 2013 main crop season. Hybrids and OPVs occupied more than 62 and 2%, respectively, whereas named and unidentified local cultivars occupied about 18% each (cf. Table 6). Thus, total MC coverage in Zambia was roughly 64%. These figures suggest that the MC adoption rate in the Zambia has not changed much over the last two decades or so, as reported elsewhere [10–12, 23], but is in sharp contrast to Morris [13] who reported 19% under hybrids and less than 1% under OPVs in 1996.

The major hybrids were PAN53, MRI624, SC513, ZMS606, and MRI634 (cf. Table 3). Together, these cultivars occupied nearly 41% of the total maize area. None of the top five (or even the top ten) ranked cultivars included any of the recently released drought-tolerant maize cultivars. The Zamseed hybrid ZMS528 was mentioned in the survey but covered 0.50% of the total area. As per previous reports [21, 29], no individual cultivar occupied appreciably more than 10% of the total area. The average number of maize cultivars grown per household in Zambia in the 2013 crop season was 1.816 (range 1–8), the highest range among all the 13 countries included in this study (cf. Table 4). Approximately 55% of the farmers surveyed in this study in Zambia planted one cultivar, 27% planted two cultivars, 10% planted three cultivars, 5% planted four cultivars, 3% planted five cultivars, with those planting 6–8 cultivars accounting for negligible percentages.

The weighted average age of maize hybrids grown in the 2013 main season in Zambia was 13 years and OPVs were about 14 years, with the combined average of 13 years. The oldest maize cultivars reported during this survey were MM604 and MM603, both released in 1984. However, the area coverage of the two combined was approximately 0.5%.

### Zimbabwe

A total of 26 cultivars were reported from Zimbabwe in the 2013 main growing season. These included 23 hybrids, two improved OPVs, and one local cultivar (cf. Table 2). Only one cultivar did not have YOR. Fifteen (58%) of the cultivars listed occupied less than 1% each of the total maize area. SC513 occupied the largest area (40%) of maize grown in Zimbabwe in 2013; other major cultivars included PAN413, PHB3253, SC403, and R201 (cf. Table 3). All together these cultivars covered 79% of the total maize area. All of them were released between 1993 and 1999.

More than 95% of the total maize area was planted to hybrids, whereas the area covered under improved OPVs was 2%, indicating a total MCs adoption of 97%, the highest in Africa. The high adoption rate of hybrids in Zimbabwe reported here is consistent with previous reports [10–13, 23].

The average number of maize cultivars grown per household in Zimbabwe in the 2013 main crop season was 1.729 (range 1–5) (cf. Table 4). Seventy-six percent of farmers in Zimbabwe planted one cultivar in the 2013 main crop season, whereas 19, 5, and 1%, respectively, planted two, three, and four cultivars.

The weighted average age of hybrids was 13 years (cf. Table 5). The OPV Hickory King (first released in 1909) was the oldest cultivar grown in 2013 in Zimbabwe. This is perhaps the oldest cultivar anywhere in Africa. It accounted for 1.69% of the total area in the current study.

### West Africa

A total of 96 cultivars were reported during this study in the four countries surveyed in WA. These comprised nine hybrids, 39 OPVs, and 48 local cultivars, in sharp contrast to EA and SA where hybrids were dominant (cf. Table 2). Information on YOR was not available for more than half of them. The average number of maize cultivars grown per household in the 2013 crop season was 1.724 (range 1–4) (cf. Table 4). Sixty-five (or 68%) of the cultivars each occupied less than 1% of the total maize area.

The total area occupied by MCs in WA was roughly 36% (consisting of less than 4% hybrids and 32% OPVs); named local cultivars covered approximately 21%, whereas unidentified local cultivars accounted for about 44% of the total area (cf. Table 6). This was a substantial improvement over the adoption rates of 10% in 1997. However, as in the other two regions, there were significant variations among countries—adoption in some countries has actually gone down.

The weighted average age of hybrids and OPVs was 13 and 17 years, respectively, with the combined average of 16 years (cf. Table 2).

### Benin

A total of 16 maize cultivars were grown in Benin in the 2013 main growing season (cf. Table 2). These included eight improved OPVs and eight local cultivars. Half of these cultivars did not have information on YOR. Fourteen of them each occupied less than 1% of the total maize area. INA, DMR, Faaba, SG2000, and TZPB SR were the top five ranked cultivars reported. Together, these cultivars covered just over 11% of the total area, indicating the dominance of unidentified cultivars (see below). All of the five cultivars were released between 1989 and 1996.

No hybrids were mentioned by farmers during the current survey. At present, the country has limited national capacity for hybrid maize development and delivery. This is further hampered by the lack of commercial seed companies in the country.^4^ The average number of maize cultivars grown per household in the 2013 main crop season was 1.162 (range 1–4) (cf. Table 4).

Improved OPVs covered about 13% of the total area, with about 29 and 59% being under known and unidentified local cultivars, respectively. The maize area under MCs reported was less than what had been reported in previous times. For example, Byerlee and Jewell [10] and Maredia et al. [11] reported 1% hybrid, and between 9 and 27% coverage of improved OPVs. Morris [13] reported 25% area under MCs. Alene et al. [16] reported approximately 3% under hybrids and 42% under MCs in 2005.

Eighty-one percent of households in Benin planted one cultivar in the 2013 main crop season, whereas 17 and 2%, respectively, planted two and three cultivars.

The weighted average age of maize cultivars in Benin was about 10 years (cf. Table 5). This by no means is an indication that maize cultivars in this country are current; rather, it indicates the paucity of R&D efforts to develop and deploy improved maize cultivars. Records show that there were no new releases between 1996 and 2007. Benin and many other countries, especially in WA, have for long heavily depended on donors for their research funding.

### Ghana

There were a total of 37 maize cultivars grown in Ghana in the 2013 main crop season (cf. Table 2). These consisted of five hybrids, 13 improved OPVs, and 19 local cultivars (cf. Table 2). No information could be found on YOR for 23 (62%) of the cultivars reported. Twenty-seven cultivars (73% of total) each covered less than 1% of the total maize area in the 2013 main crop season. With nearly 41% area coverage, Obatanpa was the dominant cultivar, followed by Aburohema, Yegboni, Appiah, and Aburotia, as distant second to fifth (cf. Table 3). Combined, these cultivars covered 48% of the total maize area grown in the 2013 main crop season. They were released between 1983 and 1992. The oldest cultivars reported in this survey were Laposta, Golden Crystal, CompW, and Comp4, all released in 1972. With the exception of the first one, all occupied less than 1% of the total area. One of the top five cultivars shown in Table 3, Aburohema, is a drought-tolerant variety released in 2010; a few others, such as Etubi, were also reported by farmers but all of them occupied less than 1% of the total area.

The total MC coverage was estimated at approximately 3% for hybrids and 50% for OPVs. Named and unidentified cultivars covered about 26 and 21%, respectively (cf. Table 5). These figures are at variance with the findings of Alene et al. [16] who reported nearly 89% coverage under MCs in 2005. Byerlee [30] reported a minimum of 16% and maximum of 48% MCs coverage in 1990.

The average number of maize cultivars grown in the 2013 main crop season in Ghana was 1.220 per household; 80% of the households planted one cultivar, whereas 18, 2, and 1%, respectively, planted two, three, and four cultivars in the 2013 main crop season.

The weighted average age of maize hybrids in Ghana was 6 years,^5^ whereas OPVs were 24 years old; the weighted overall average age of all varieties was nearly 23 years.

### Mali

With a total of ten cultivars reported, Mali had the least number of maize cultivars grown in the 13 countries in the 2013 main crop season. These included eight improved OPVs and two local cultivars (cf. Table 2); no hybrids were mentioned by farmers in this survey. Information on YOR could not be found for two of the cultivars reported. None of the cultivars occupied less than 1% of the total area. The average number of maize cultivars grown per household in the 2013 main crop season was 1.149 (range 1–3) (Table 4), the lowest among all countries.

With nearly 24% of the total area coverage, Sotubaka was the dominant cultivar (cf. Table 3); this was followed by Dembanyuman (or Obatanpa), Burkina, Nieleni, Zangreni, and N’Boni, each of which covered between nearly 3 and 13%. The top five ranked cultivars covered 47% of the total maize area. These were released between 1985 and 1998 (cf. Table 3).

MCs (all improved OPVs) covered 51% of the total area, whereas named and unidentified local cultivars occupied approximately 13 and 36%, respectively (cf. Table 6). These figures are consistent with reports by Byerlee and Jewell [10], Maredia et al. [11], and Morris [13]. However, they are at variance with what Alene et al. [16] reported for the 2005 season—MC coverage of nearly 86%.

Eighty-two percent of households in Mali planted one cultivar, whereas 16 and 1%, respectively, planted two and three cultivars.

The weighted average age of maize cultivars grown in Mali in 2013 was nearly 18 years (cf. Table 5). The oldest cultivar grown during this survey year was TZE SR W (released in 1983); it covered less than 2% of the total area.

### Nigeria

The total number of maize cultivars recorded during the current survey in Nigeria was 33. These comprised four hybrids, ten OPVs, and 19 local cultivars (cf. Table 2). Information on YOR for 19 (58%) of the cultivars could not be found; 24 (73%) of the cultivars each occupied less than 1% of the total area. Oba Super9, Ba Hausa, EVDT 99, 3DT Com, and Yar Masara were the top five ranked cultivars (cf. Table 3). These covered nearly 23% of the total maize area in Nigeria. All, except Yar Masara, were drought-tolerant cultivars released in 2009. Other such cultivars included Sammaz37, TZE Comp3, Notore, Samaru15, and 2009 EVDT, all released between 2008 and 2011.

Approximately 12% of the maize area in this country was under hybrids, with improved OPVs covering close to 15% of the total area; named and unidentified local cultivars covered roughly 15 and 58% of the total area, respectively (cf. Table 6). Thus, the total area under MCs was about 27%. These figures are far lower than what had been previously reported by various authors (e.g., [10, 11, 13]). Alene et al. [16] reported that approximately 61% of the total maize area in Nigeria was covered under MCs in 2005.

The average number of maize cultivars grown per household in Nigeria in the 2013 crop season was 3.363 (range 2–4), the highest among all countries (Table 4). Seventy-eight percent of households planted two maize cultivars in Nigeria in the 2013 main crop season, whereas 21 and 1%, respectively, planted three and four cultivars.

The average age of maize cultivars in Nigeria was approximately 15 years for hybrids, 12 years for OPVs, and 13 years for all cultivars (cf. Table 5). The oldest cultivar recorded in this survey was the OPV SYN1 COM, released in 1963; it covered less than 1% of the total area.

## Discussion

This study has helped to identify maize cultivars that are currently grown by smallholder farmers in SSA and to estimate their adoption level. Such information provides useful tools for decision making, in addition to helping in the identification of cultivars that should be targeted for replacement. The regional differences in all the parameters measured in this study may be explained by the variations in the level of investment in maize R&D among the regions and countries within them. For example, there had been relatively rapid and consistent growth of cultivar releases in SA and EA starting in the late 1990s, whereas this was not the case for WA, perhaps with the exception of Nigeria. There was a good level of cultivar releases in this region up to the late 1990s, but it came to almost a complete halt in later years, following the end of WECAMAN (West and Central Africa Maize Network). Many African countries, especially in WA, have for long heavily depended on donors for their research funding. Benin, Ghana, Malawi, Mali, and Mozambique had almost no modern cultivars released from 1996 up to 2011. The preponderance of old and unidentified cultivars and relatively less complete database on maize R&D could also be attributed to the low level of investment.

The weighted average age of maize cultivars for all countries reported here is older than what is generally practiced in the USA or other regions such as South America and Asia. Approximately eight out of the top ten cultivars listed for all countries in this study were released in or before 2005; the only exception was Nigeria, where half of the top ten cultivars reported were released after 2009.

On the other hand, it is interesting to note that more recent releases of drought-tolerant cultivars were mentioned by farmers during this study in several countries, even though their area coverage was low. Examples included Longe 10H in Uganda, ZMS528 in Zambia, PAN 4M-19 in Kenya, Vumilia 1H in Tanzania, ZM309 in Mozambique, Sammaz37 and 2009 EVDT in Nigeria, DT R W Co in Benin, Aburohema and Etubi in Ghana.

This study has also demonstrated that an average African farm household grew 1.781 (range 1–8) maize cultivars in the 2013/2014 crop season. The purpose behind planting more than one cultivar might vary from culinary value to security against crop loss due to adversity (such as drought, pest and disease outbreaks, and others). Whatever the reason behind planting multiple maize cultivars per household, this practice contributes to increased biodiversity of the maize farming system in Africa. Smale et al. [31] reported that 99% of households in Malawi preferred local maize for home consumption. De Groote et al. [24] observed that criteria for variety choice of maize farmers in Ethiopia and Tanzania included taste, digestibility, nutritional quality, stover biomass, and quality for animal feed, among other traits. A recent study by Lunduka et al. [32] indicated that farmer interest in a diversity of seed attributes partly explained adoption plateaus for modern maize cultivars in Malawi. Their sampled farmers expressed strong preferences for particular traits found in local maize varieties: ease of storage, high poundability, high flour–grain ratio, and favorable taste. OPVs were also attractive to many of the sampled farmers, particularly those who valued early maturity and faced binding cash constraints. Farmers also revealed strong interest to grow hybrid maize for yield and drought tolerance. For the case of rice in the Ivory Coast, Dalton [33] documented that the failure to incorporate both production and consumption traits and the over-emphasis on yield in national breeding efforts resulted in biased and inappropriate varietal promotions and, subsequently, low adoption of new varieties of upland rice by farmers. Similarly, households in Ethiopia have been observed to grow local maize side-by-side improved varieties—the former for home consumption and the latter for market.^6^ It is highly plausible that this situation is common in many other African countries. This suggests that maize breeding efforts should consider a diversity of traits beyond stress tolerance and grain yield to encompass the range of production, processing, and consumption attributes that are valued by farmers.

The fact that 28 of the cultivars listed were each grown has been successfully commercialized in multiple countries begs an obvious question on our breeding strategies. That is, do we focus on a limited number of maize cultivars that have wider adaptation, or do we concentrate on cultivars that are suited to limited agroecologies within each country? Which approach would yield the optimum results for your resources and efforts? What would be the implications of either strategy?

Number of cultivars released and adoption rate have often been used as a measure of success for maize technologies in the past; however, experience from the literature suggests that high adoption does not necessarily always translate into productivity growth. Number of cultivars released is only one part of the equation in productivity growth. We witness that sustained adoption and subsequent productivity gains depend largely on conducive government policy [24, 34–37] that would enable increased national government investment in agriculture, availability of inputs (seed and fertilizer) at affordable prices, a strong extension system, and market outlets for products.

**Fig. 4:**
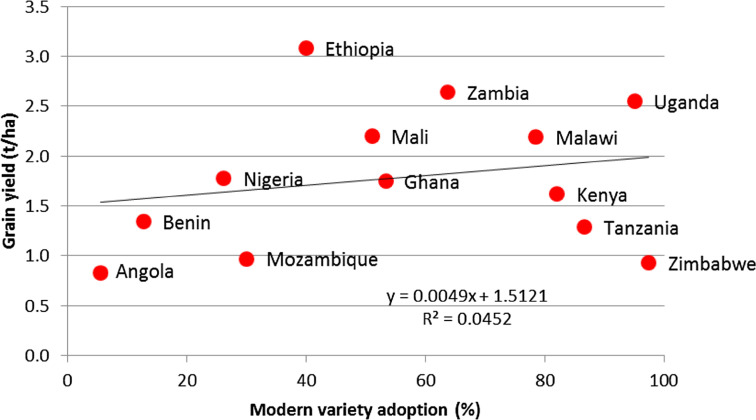
Relationship between modern maize cultivar adoption and grain yield in the 13 surveyed African countries in the 2013/2014 main crop season

Figure 4 illustrates this point very clearly for Zimbabwe, which has the highest adoption rate (97%) of MCs on the continent. However, its 2010–2013 average yield was hardly higher than that of Angola, which has among the lowest adoption rates (6%). Furthermore, the national average adoption rate for Ethiopia has been estimated at 40% [1], but its average maize yield was roughly 3.4 MT/ha, superior to eight of the countries whose adoption rates are much higher. Obviously, adoption of improved cultivars is an important step toward achieving improved, but the above examples illustrate that adoption alone is not enough to attain this goal. African governments need to enhance their input use and strengthen infrastructure and institutions in order to improve their food security situation in their countries.

## Conclusions

The substantial differences among regions and countries for almost all parameters measured in this study suggest that interventions toward mitigating the constraints need to be tailored to the objective situations of the regions and countries. Meanwhile, it would be interesting to find out why farmers still grow decades old cultivars—especially in situations where they have options. What are the characteristics of the maize cultivars that attract them to those cultivars? We certainly know that it is not yield; nor is it their tolerance/resistance to abiotic and biotic stresses, as is the case with Hickory King in Zimbabwe.

The variety turnover time for hybrid maize in the USA was shortened from 8 years in the early 1990s to about 5 years in 2010; it could even be three to 4 years now.^7^ The variety turnover time in parts of Mexico, Brazil, and Argentina ranges from three to 4 years in the tropics (where maize is considered a high value crop) to 5–7 years in the subtropics.^8^ The estimated current variety turnover time in Asia is 5–6 years.^9^ Given 2–3 years for new cultivars from release to seed production, promotion, and diffusion in SSA, it is fair to say that all cultivars in Africa that are older than 10 years should be eligible for replacement. Within those, cultivars occupying the largest area should be given the priority to be replaced. In principle, it is important to use the most recent cultivars available, as these are tolerant or resistant to multiple stresses and more resilient in light of climate change and ever erratic rainfall patterns. Fortunately, there is no paucity of new cultivars to replace the old ones (www.stma.cimmyt.org).

## Data Availability

The data supporting the results of this article are included within the article; detailed list of maize cultivars reported from each country will soon be on our Web site.

## Footnotes

1 FAOSTAT for 2014.

2 Refers to non-recycled hybrids and improved OPVs that have not been recycled more than three seasons.

3 Derived from Obatanpa, an OPV widely grown in Ghana, Mali and Uganda; also reported from Nigeria and Zambia.

4 Five new seed companies were registered in 2015.

5 Please note that there was only one hybrid with information on YOR.

6 Tesfaye Kumsa (pers. com.), Director of Anno Agro-Industry Seed Company, Bako, Ethiopia, September 2015.

7 Aline O’Connor (pers. com.), Director, Agri Experience Ltd., March 2017.

8 Arturo Silva Hinojosa (pers. com.), Maize Seed Specialist, CIMMYT-Mexico, March 2017.

9 Ramaiah Sadananda Ajanahalli (pers. com.), Maize Seed Specialist, CIM-MYT-Nepal, March 2017.

## Abbreviations

AGRITEX: Agricultural Research and Extension (Zimbabwe);
AR&D: Agricultural Research and Development;
B&MGF: Bill and Melinda Gates Foundation;
CGIAR: Consultative Group on International Agricultural Research;
CIMMYT: International Center for Maize and Wheat Improvement (Spanish acronym);
CRS: Christian Relief Services;
CSIR: Council for Scientific and Industrial Research;
DTMA: Drought Tolerant Maize for Africa;
EA: eastern Africa;
EIAR: Ethiopian Institute of Agricultural Research;
FAOSTAT: Food and Agricultural Organization (statistical database);
IER: Institute of Rural Economy (French acronym, Mali);
IIA: Institute of Agricultural Research (Portuguese acronym, Angola);
IIAM: Institute of Agricultural Research for Mozambique (Portuguese acronym);
KALRO: Kenya Agricultural and Livestock Research Organization;
MC: modern cultivar;
MT: metric ton;
NARO: National Agricultural Research Organization (Uganda);
NGO: non-governmental organization;
OPV: open-pollinated variety;
R&D: research and development;
SA: southern Africa;
SARI: Savanna Agricultural Research Institute (Tamale, Ghana);
SARI: Selian Agricultural Research Institute (Arusha, Tanzania);
SSA: sub-Saharan Africa;
STMA: Stress Tolerant Maize for Africa;
USAID: United States Agency for International Development;
WA: western Africa;
WECAMAN: Western and Central Africa Maize Network;
YOR: year of release.

## Authors’ information

Tsedeke Abate is leader for STMA and CIMMYT’s Maize Seed Systems for Africa, based in Nairobi, Kenya; Monica Fisher was formerly agricultural economist at CIMMYT in Addis Ababa, Ethiopia—currently she is assistant professor of economics at Oregon State University, USA; Tahirou Abdoulaye is senior economist at IITA, Ibadan, Nigeria; Girma T. Kassie was formerly agricultural economist at CIMMYT in Harare, Zimbabwe, and is currently with ICARDA, based in Addis Ababa; Rodney Lunduka is agricultural economist at CIMMYT in Harare; Paswel Marenya is agricultural economist based at CIMMYT in Addis Ababa; Woinishet Asnake is research associate at CIMMYT in Addis Ababa.

## References

[1] Abate T, Shiferaw B, Menkir A, Wegary D, Kebede Y, Tesfaye K, Kassie M, Bogale G, Tadesse B, Keno T. Factors that transformed maize productivity in Ethiopia. Food Secur. 2015. doi:10.1007/s12571-015-0488-z.

[2] Shiferaw B, Prasanna B, Hellin J, Bänziger M. Crops that feed the world: past successes and future challenges to the role played by maize in global food security. Food Secur. 2011;3:307–27.

[3] Louwaars NP, De Boef W, Edeme J. Integrated seed sector development in Africa: a basis for seed policy laws. J Crop Improv. 2013;27:186–214.

[4] Ragasa C, Dankyi A, Acheampong P, Wiredu AN, Chapo-to A, Asamoah M, Tripp R. Patterns of adoption of maize technologies in Ghana Ghana Strategy Support Program working paper 36 2013; IFPRI.

[5] Fisher M, Abate T, Lunduka RW, Asnake W, Alemayehu Y, Madulu BR. Drought tolerant maize for farmer adaptation to drought in sub-Saharan Africa: determinants of adoption in eastern and southern Africa. Clim Change. 2015. doi:10.1007/s10584-015-1459-2.

[6] Miracle MP. The introduction and spread of maize in Africa. J Afr Hist. 1965;6:39–55.

[7] Magorokosho C. The Impact of farmers’ selections on adapting maize landraces to diverse agro-ecological conditions in Zimbabwe, Zambia, and Malawi, and genetic gain and diversity trends in maize varieties released and grown in Zimbabwe from 1900 to 2004 Texas A&M University; 2006.

[8] Mason NM, Ricker-Gilbert J. Disrupting demand for commercial seed: input subsidies in Malawi and Zambia. World Dev. 2012;45:75–91.

[9] Brennan JP, Byerlee D. The rate of crop varietal replacement on farms: measures and empirical results for wheat. Plant Var Seeds. 1991;4:99–106.

[10] Byerlee D, Jewell D. The technological foundation of the revolution In: Byerlee D, Eicher CK, editors. Africa’s emerging maize revolution. Boulder: Lynne Rienner Publishers; 1997 p. 127–43.

[11] Maredia M, Byerlee D, Pee P. Impacts of food crop improvement research in Africa SPAAR Occasional Papers Series, No. 1, Special Program for African Agricultural Research, Washington DC: World Bank; 1998.

[12] Hassan RM, Mekuria M, Mwangi W. Maize breeding research in eastern and southern Africa: Current status and impacts of past investments made by the public and private sectors 1966–97. Mexico, D.F.: CIMMYT; 2001.

[13] Morris ML. Impacts of international maize breeding research in developing countries, 1966–98. Mexico, D.F.: CIMMYT; 2002.

[14] Spielman DJ, Alemu D, Kelemework D. Seeds, fertilizer, and agricultural extension in Ethiopia In: Dorosh P, Rashid S, editors. Food and agricultural policies in Ethiopia: progress and challenges. Philadelphia: University of Pennsylvania Press; 2013.

[15] Khonje M, Manda J, Alene AD, Kassie M. Analysis of adoption and impacts of improved maize varieties in eastern Zambia. World Dev. 2014;66:695–706.

[16] Alene AD, Menkir A, Ajala SO, Badu-Apraku B, Olanrewaju AS, Manyong VM, Ndiaye A. The economic and poverty impacts of maize research in West and Central Africa. Agric Econ. 2009;40:535–50.

[17] Smale M, Byerlee D, Jayne T. Maize revolutions in sub-Saharan Africa. Development Research Group, Agriculture and Rural Development Team, Policy Working Paper 5659, Washington, DC: World Bank; 2011.

[18] Lyimo S, Mduruma Z, De Groote H. The use of improved maize varieties in Tanzania. Afr J Agric Res. 2014;9:643–57.

[19] Smale M, Olwande J. Demand for maize hybrids and hybrid change on smallholder farms in Kenya. Agric Econ. 2014;45:1–12.

[20] Walker T, Alene A, Ndjeunga J, Labarta R, Yigezu Y, Diagne A, Andrade R, Andriatsitohaina R, De Groote H, Mausch K, Yirga C, Simtowe F, Katungi E, Jogo W, Jaleta M, Pandey S. Measuring the effectiveness of crop improvement research in sub-Saharan Africa from the perspectives of varietal output, adoption, and change: 20 crops, 30 countries, and 1150 cultivars in farmers’ fields. Rome: CGIAR Independent Science and Partnership Council Secretariat; 2014.

[21] De Groote H, Gitonga Z, Smale M, Kasuta M, Asare-Marfo D, Sonder K, Birol E. Adoption and diversification of modern maize varieties in Zambia in 2011. A baseline report prepared for HarvestPlus; Mexico, D.F.: CIMMYT; 2012.

[22] Beshir B, Wegary D. Determinants of smallholder farmers’ hybrid maize adoption in the drought prone central rift valley of Ethiopia. Afr J Agric Res. 2014;9:1334–43.

[23] Langyintuo AS, Mwangi W, Diallo AO, MacRobert J, Dixon J, Bänziger M. An analysis of the bottlenecks affecting the production and deployment of maize seed in eastern and southern Africa. Harare, Zimbabwe: CIMMYT; 2008.

[24] De Groote H, Dema G, Sonda GB, Gitonga ZM. Maize for food and feed—the farmers’ perspective. Field Crop Res. 2013;153:22–36.

[25] Jaleta M, Yirga C, Kassie M, De Groote H, Shiferaw B. Knowledge, adoption and use intensity of improved maize technologies in Ethiopia. Invited paper presented at the 4th International Conference of the African Association of Agricultural Economists, Hammamet, Tunisia; 2013.

[26] De Groote H, Gitonga Z, Mugo S, Walker T. Assessing the effectiveness of maize and wheat improvement from the perspectives of varietal output and adoption in eastern and southern Africa. CIMMYT; 2016.

[27] Westengen OT, Ring KH, Berg PR, Brysting AK. Modern maize varieties going local in the semi-arid zone in Tanzania. BMC Evol Biol. 2014. doi:10.1186/1471-2148-14-1. PMC3890540 24382122

[28] Manuvanga K, Kissungo D, Chindongo IP, Morais O, Nzambi VK. Adoption of drought tolerant maize in Angola. Huambo: IIA; 2013.

[29] Smale M, Simpungwe E, Birol E, Kassie GT, De Groote H, Mutale R. The changing structure of the maize seed industry in Zambia: prospects for orange maize. Agribusiness. 2015. doi:10.1002/agr.21384.

[30] Byerlee D. Modern varieties, productivity, and sustainability: recent experiences and emerging challenges. Mexico, D.F.: CIMMYT; 1994.

[31] Smale M, Heisey PW, Leathers HD. Maize of the ancestors and modern varieties: the microeconomics of high-yielding variety adoption in Malawi. Econ Dev Cult Change. 1995;43:351–68.

[32] Lunduka R, Fisher M, Snapp S. Could farmer interest in a diversity of seed attributes explain adoption plateaus for modern maize varieties in Malawi? Food Policy. 2012;37:504–10.

[33] Dalton TJ. A household hedonic model of rice traits: economic values from farmers in West Africa. Agric Econ. 2004;31:149–59.

[34] De Groote H, Owuor G, Doss C, Ouma J, Muhammad L, Danda K. The maize green revolution in Kenya revisited. e-J Agric Dev Econ. 2005;2:32–49.

[35] Abate T, Shiferaw B, Gebeyehu S, Amsalu B, Negash K, Assefa K, Eshete M, Aliye S, Hagmann J. A systems and partnership approach to agricultural research for development: lessons from Ethiopia. Outlook Agric. 2011;40:213–20.

[36] Abate T, Orr A. Research and development for tropical legumes: towards a knowledge-based strategy. J SAT Agric Res. 2012;10:1–12.

[37] Smale M, Byerlee D, Jayne T. Maize revolutions in sub-Saharan Africa. In Otsuka K, Larson DF, editors An African green revolution: finding ways to boost productivity on small farms. 2013. doi:10.1007/978-94-007-5760-8_8.

